# GHRH + arginine test and body mass index: do we need to review diagnostic criteria for GH deficiency?

**DOI:** 10.1007/s40618-023-02081-9

**Published:** 2023-04-16

**Authors:** V. Gasco, D. Cuboni, E. Varaldo, F. Bioletto, A. M. Berton, C. Bona, N. Prencipe, E. Ghigo, M. Maccario, S. Grottoli

**Affiliations:** Department of Medical Science, Division of Endocrinology, Diabetes and Metabolism, University of Turin, Turin, Italy

**Keywords:** GHRH + arginine, GH deficiency, Obesity, Diagnosis, Sensibility, Specificity

## Abstract

**Introduction:**

The proportion of patients with low GH response to provocative tests increases with the number of other pituitary hormone deficiencies, reason why in panhypopituitary patients GH stimulation tests may be unnecessary to diagnose GH deficiency (GHD)

**Purpose:**

To re-evaluate the diagnostic cut-offs of GH response to GHRH + arginine (ARG) test related to BMI, considering the patients’ pituitary function as the gold standard for the diagnosis of GHD.

**Methods:**

The GH responses to GHRH + ARG were studied in 358 patients with history of hypothalamic-pituitary disease. GHD was defined by the presence of at least 3 other pituitary deficits (n = 223), while a preserved somatotropic function was defined by the lack of other pituitary deficits and an IGF-I SDS ≥ 0 (n = 135). The cut-off with the best sensitivity (SE) and specificity (SP), was identified for each BMI category using the ROC curve analysis. To avoid over-diagnosis of GHD we subsequently searched for the cut-offs with a SP ≥ 95%.

**Results:**

The best GH cut-off was 8.0 μg/l (SE 95%, SP 100%) in lean, 7.0 μg/l (SE 97.3%, SP 82.8%) in overweight, and 2.8 μg/l (SE 84.3%, SP 91.7%) in obese subjects. The cut-off with a SP ≥ 95% was 2.6 μg/l (SE 68.5%, SP 96.6%) in overweight and 1.75 μg/l (SE 70.0%, SP 97.2%) in obese subjects.

**Conclusions:**

This is the first study that evaluates the diagnostic cut-offs of GH response to GHRH + ARG related to BMI using a clinical definition of GHD as gold standard. Our results suggest that with this new approach, the GHRH + ARG cut-offs should be revised to avoid GHD over-diagnosis.

## Introduction

Adult GH deficiency (GHD) is a pathological condition that may occur during childhood or adult life, resulting from genetic or congenital disorders of pituitary development, as well as be secondary to central nervous system (CNS) injury including tumors, surgery, trauma, radiation or infiltrative diseases [1, 2]. This condition is characterized by altered glucose and lipid metabolism, derangement of body composition, premature atherosclerosis, osteoporosis, impaired quality of life, and increased mortality [3–6]. GH replacement therapy improves most of these abnormalities [7, 8]. However, to date, a reduction in cardiovascular mortality during GH treatment remains to be demonstrated, and considering the high cost of the replacement treatment [9] and its potential long-term risks, it is essential to establish the correct diagnosis so that appropriate GH replacement is offered only to adults who are truly GH-deficient.

Adult GHD diagnosis depends on the demonstration of a reduced peak serum GH level in response to one or more GH stimulation tests [2, 10, 11].

Obesity is a state of functional GHD, with decreased spontaneous secretion, pulses, and half-life of GH [12–14]. Moreover, a decreased GH responsiveness to all stimulation tests has been demonstrated in obesity as well as in subjects with abdominal adiposity [15–18]. Therefore, one of the warnings in interpreting the results of most GH stimulation tests in adults is the high prevalence of obesity in the general population, as well as in patients with acquired hypothalamic-pituitary disease [19].

Insulin tolerance test (ITT) is the gold standard for the diagnosis of adult GHD. At the same time, GH-releasing hormone (GHRH) in combination with GH-releasing peptide-6 (GHRP-6) or arginine (ARG) are recognized as equally faithful tests [2, 10, 11], and glucagon stimulation is considered of good diagnostic value [20]. To date all these tests have clear cut-offs and normative BMI based reference values [21–24]. Recently, the macimorelin test has been demonstrated to be safe, effective, highly reproducible [25, 26] and therefore in December 2017 the FDA (https://www.accessdata.fda.gov/drugsatfda_docs/label/2017/205598s000lbl.pdf) ad in January 2019 the EMA (https://www.ema.europa.eu/en/medicines/human/EPAR/ghryvelin-previously-macimorelin-aeterna-zentaris) approved oral macimorelin for use as a diagnostic test for adult GHD.

However, it should be considered that, unlike previous tests, to date macimorelin has no specific cut-offs for overweight or obese patients. Lastly, it should be considered that all previous studies have evaluated the GH response to various stimulus tests by comparing the response of patients who are certainly GHD with normal subjects [22–24, 27, 28] and/or by using another stimulation test as gold standard [21, 25–29]. This approach is exposed to possible bias. In particular, comparing patients with normal subjects, even if matched by sex, age and BMI, is at risk of overestimating the diagnostic accuracy of the cut-offs thus identified [30]. Moreover, comparing a particular test with another diagnostic test, considered the gold standard, is at risk of overestimating or underestimating the diagnosis in a similar way to what the gold standard itself does. The proportion of patients with low GH response to provocative tests increases with the number of other pituitary hormone deficiencies and several studies involving panhypopituitary patients have shown that under certain circumstances GH stimulation tests may be unnecessary to diagnose GHD [2, 10, 11]. Aim of this study was to re-evaluate the diagnostic cut-offs of GH response to GHRH + ARG test related to BMI. To this aim the patients’ pituitary function was considered as the gold standard for the diagnosis or exclusion of GHD.

## Materials and methods

### Patient selection and data collection

**Fig. 1:**
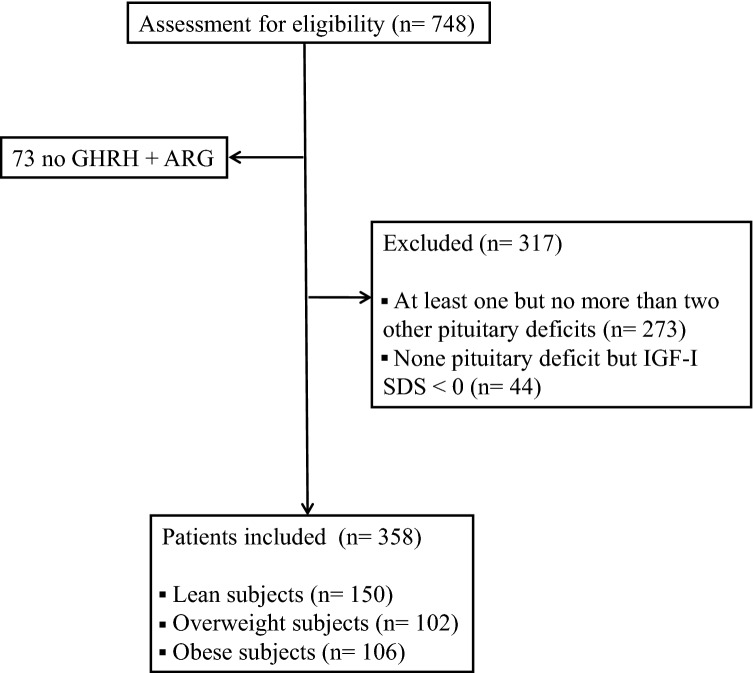
Consort diagram of the study population

We retrospectively analyzed the data of 748 patients with history of pituitary disease, referred to the Neuroendocrinology Clinic of our Center from 01.01.2016 to 31.12.2018 for the evaluation of GH secretion. The patients’ pituitary function was considered as the gold standard for the diagnosis or exclusion of GHD; in particular GHD was defined by the presence of at least 3 other pituitary deficits, while a preserved somatotropic function was defined by the lack of other pituitary deficits with an IGF-I standard deviation score (SDS) ≥ 0. Seventy-three patients never performed a GHRH + ARG test and were therefore excluded. Two hundred and seventy-three patients were excluded because they presented at least one but no more than two other pituitary deficits except the assumed GHD; 44 patients were excluded because, even if they did not have any pituitary deficit, they presented an IGF-I SDS < 0. A consort flow diagram of the study population is outlined in Fig. 1. Therefore, the final study population consisted in 358 patients (218 M, 140 F; age [mean ± SD]: 45.1 ± 17.2 years; BMI: 27.2 ± 5.8 kg/m^2^), subdivided in relation to BMI in 150 normal weight, 102 overweight, and 106 obese subjects. Patients’ clinical characteristics are reported in Table 1. We studied the GH response to GHRH (1 µg/kg i.v. at 0 min) + ARG (0.5 g/kg infused i.v. from 0 to + 30 min) (sampling every 15 min from + 30 to + 60 min) as previously described [24]. IGF-I levels were measured in all patients. All subjects gave their informed consent to the processing of their data. The study was approved by the local Ethics Committee and was in accordance with the principles of the Declaration of Helsinki.

**Table 1:** Clinical features of both the whole cohort of 358 patients with history of hypothalamic-pituitary disease and subdivided according to the presence (GHD) or absence (noGHD) of adult growth hormone deficiency defined by a clinical point of view

	All study population (n=358)	GHD (n=223)	noGHD (n=135)	p
**Gender**				
Male subjects (%)	218 (60.9)	142 (63.7)	76 (56.3)	0.20
Female subjects (%)	140 (39.1)	81 (36.3)	59 (43.7)	
**Age (mean±SD) yrs**	45.1 ± 17.2	47.5 ± 16.2	41.0 ± 18.1	<0.001
**BMI (mean±SD) kg/m**^**2**^	27.2 ± 5.8	27.8 ± 5.6	26.3 ± 6.0	0.01
Lean subjects (%)	150 (41.9)	80 (35.9)	70 (51.8)	0.02
Overweight subjects (%)	102 (28.5)	73 (32.7)*	29 (21.5)*	
Obese subjects (%)	106 (29.6)	70 (31.4)*	36 (26.7)*	
**Peak GH to GHRH +ARG test (mean±SD) µg/l**	13.8 ± 24.4	2.1 ± 3.0	34.8 ± 36.1	< 0.001
**IGF-I (mean±SD) µg/l**	123.1 ± 91.0	79.2 ± 42.6	240.0 ±83.5	< 0.001
**IGF-I SDS**	− 0.7 ± 1.3	− 1.3 ± 0.79	0.92 ± 0.69	< 0.001
**Pathology (%)**				
Pituitary adenomas	157 (43.9)	121 (54.3)*	36 (26.7)*	
Craniopharingioma + Rathke’s cleft cyst	33 (9.2)	32 (14.3)*	1 (0.7)*	
Primary + secondary (due to Sheehan syndrome) empty sella	69 (19.3)	51 (22.9)*	18 (13.3)*	0.04
Idiophatic CO-GHD	17 (4.7)	0 (0)	17 (12.6)	
Tramatic brain injury + ESA	54 (15.1)	6 (2.7)*	48 (35.6)*	
Other	28 (7.8)	13 (5.8)	15 (11.1)	
**Treatment of the pituitary pathology (%)**				
Neurosurgery alone	155 (43.3)	105 (47.1)*	50 (37.0)*	< 0.001
Radiotherapy alone	6 (1.7)	3 (1.3)	3 (2.3)	
Neurosurgery + radiotherapy	56 (15.6)	51 (22.9)*	5 (3.7)*	
None	141 (39.4)	64 (28.7)	77 (57.0)	

*p < 0.001 GHD vs noGHD

### Analytical methods

Serum GH levels (μg/l) were measured in duplicate by IRMA method (IRMA GH, Beckman Coulter, Czech Republic). The IRMA assay of GH is a sandwich-type assay. The kit utilizes mouse monoclonal antibodies directed against two different epitopes of the molecule. The antibodies recognize the 22 kDa monomer, the dimer and GH bound to its binding protein. The calibrators are balanced out on the international standard WHO 2nd IS 98/574 in human serum. The sensitivity of the assay was 0.033 µg/l. The inter- and intra-assay coefficients of variation (CV) were 9.0–14.0% and 2.4–6.5%, respectively. Serum IGF-I levels (μg/l) were measured in duplicate by RIA method (SM-C-RIA-CT, DIAsource Immuno Assays, Belgium) after acid–ethanol extraction to avoid interference by binding proteins. The sensitivity of the method was 0.25 μg/l. The inter- and intra-assay CV were 6.8–14.9% and 4.5–7.0%, respectively. IGF-I levels are expressed both as an absolute value and as a SDS of the mean normal value. The SDS for each subject was calculated in accordance with the published normality data on a population of 547 healthy Italian subjects [31]. All samples from an individual subject were analysed together.

### Statistical analysis

Statistical analysis was performed using STATA 17 (StataCorp, College Station, Texas, USA).

Baseline patients’ characteristics were summarized using mean and standard deviation (SD) for continuous data and percent values for categorical data. Between-group differences were evaluated by Student t test or by Chi-squared test, as appropriate.

We tried to identify the best GH cut-off to GHRH + ARG for the diagnosis of GHD using the Receiver-Operating Characteristic (ROC) curve analysis. The best cut-off in a ROC curve is the closest point to that with the theoretical maximum of both sensibility (SE) and specificity (SP). In order to avoid over-diagnosis of GHD we subsequently searched for the cut-offs with a SP ≥ 95%. The diagnostic cut-off points were calculated for the lean (BMI < 25 kg/m^2^), overweight (BMI 25–29.9 kg/m^2^), and obese (BMI ≥ 30 kg/m^2^) groups. For each identified cut-off we also calculated the positive predictive value (PPV), the negative predictive value (NPV), the positive likelihood ratio (LHR +), and the negative likelihood ratio (LHR-). A LHR + greater than 1 indicates that a positive result to the test is associated with the disease. A LHR- less than 1 indicates that a negative result to the test is associated with absence of the disease. Tests where the likelihood ratios lie close to 1 have little practical significance, as the post-test probability (odds) is little different from the pre-test probability, and as such is used primarily for diagnostic purposes, and not screening purposes. When the LHR + is greater than 5 or the LHR- is less than 0.2 (i.e.1/5), then they can be applied to the pre-test probability of a patient having the disease tested for to estimate a post-test probability of the disease state existing [32]. The accuracy of the identified cut-offs was defined as the probability of a person who has the disease and of a person who does not has the disease testing positive and negative, respectively.

## Results

Based on patients’ pituitary function 223 patients were defined GHD (142 M, 81 F) and 135 patients were defined as not affected by GHD (noGHD; 76 M, 59 F). The two groups differed both for age (GHD vs noGHD: 47.5 ± 16.2 vs 41.0 ± 18.1 yrs, p < 0.001) and BMI (GHD vs noGHD: 27.8 ± 5.6 vs 26.3 ± 6.0 kg/m^2^, p = 0.01) (Table 1). An hypothalamic-pituitary tumor was more frequent in GHD than in noGHD patients, while a previous diagnosis of idiopathic childhood onset GHD and a history of traumatic brain injury or subarachnoid haemorrhage were more frequent in noGHD than in GHD patients (Table 1). Previous neurosurgical treatment alone or associated with radiotherapy was more frequent in GHD patients (Table 1), while a “wait and see” approach was more common in noGHD (Table 1).

IGF-I levels in noGHD were higher than in GHD when expressed both as mean and SDS levels (240.0 ± 83.5 μg/l vs 79.2 ± 42.6 μg/l, p < 0.001; 0.92 ± 0.69 vs − 1.3 ± 0.79, p < 0.001) (Table 1), with a clear overlap in the two groups both in IGF-I levels (IGF-I range in noGHD vs GHD: 118–539 μg/l vs 8–268 μg/l) and IGF-I SDS values (IGF-I SDS range in noGHD vs GHD: 0.01–2.98 vs − 2.71–1.85).

The mean GH response to GHRH + ARG in noGHD patients was higher than that recorded in GHD (34.8 ± 36.1 μg/l vs 2.1 ± 3.0 μg/l, p < 0.001) (Table 1).

**Fig. 2:**
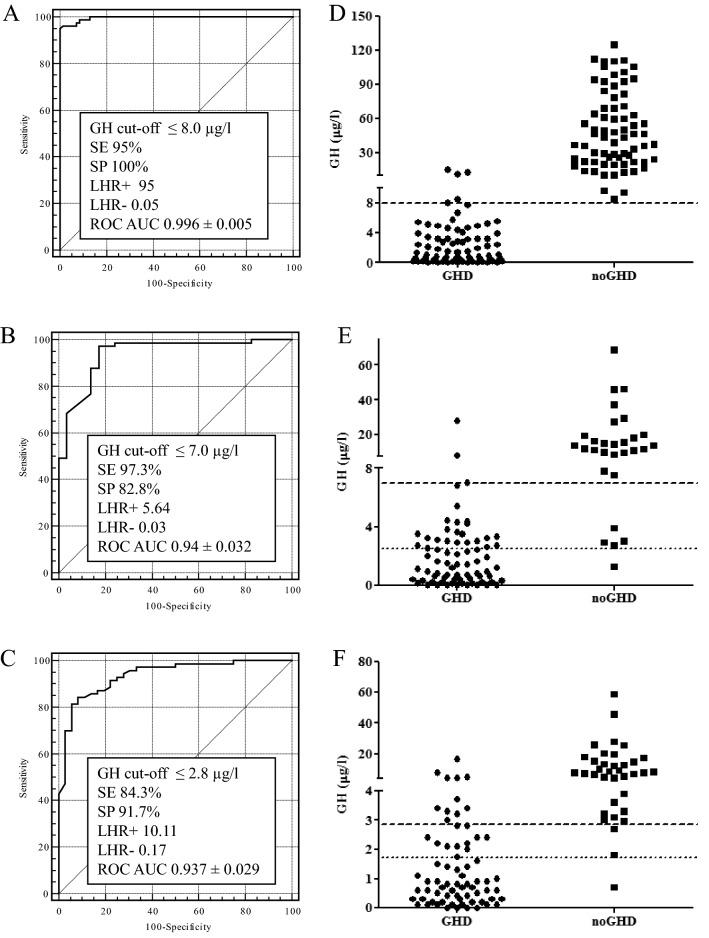
ROC analysis to identify the best GH cut-off to GHRH + ARG in lean (**A**), overweight (**B**) and obese (**C**) patients. Individual value of peak GH to GHRH + ARG compared both to the GH cut-off with the best SE and the best SP (dashed line) and to the GH cut-off with a SP ≥ 95% (dotted line) according to ROC analysis in lean (**D**), overweight (**E**) and obese (**F**) patients (*SE* sensibility, *SP* specificity, *LHR* + positive likelihood ratio, *LHR-* negative likelihood ratio, *ROC AUC* area under the ROC curve)

The best GH cut-offs to GHRH + ARG were: (i) 8.0 μg/l in lean subjects with a SE value of 95% and a SP value of 100%; (ii) 7.0 μg/l in overweight subjects with a SE value of 97.3% and a SP value of 82.8%; (iii) 2.8 μg/l in obese subjects with a SE value of 84.3% and a SP value of 91.7%, respectively (Fig. 2, Table 2).

**Table 2:** Sensibility (SE), specificity (SP), positive predictive value (PPV), negative predictive value (NPV), area under the ROC curve (ROC AUC) ± standard deviation (SD), positive likelihood ratio (LHR+), negative likelihood ratio (LHR-), and the diagnostic accuracy of the cut-off limits to GHRH + ARG identified with the ROC analysis in lean, overweight and obese patients and compared with the currently recognized cut-offs

References	BMI (kg/m^2^)	Cut-off (µg/l)	SE (%)	SP (%)	PPV (%)	NPV (%)	LHR+	LHR-	ROC AUC (mean±SD)	Diagnostic accuracy (%)
This study: best SE and SP and SP ≥ 95%	< 25	≤ 8.0	95.0	100	100	94.6	95	0.05	0.996 ± 0.005	97.3
[24]		≤ 11.5	97.5	92.9	94.0	97.0	13.73	0.03		95.3
This study: best SE and SP	25-29.9	≤ 7.0	97.3	82.8	93.4	92.3	5.64	0.03	0.940 ± 0.032	93.1
This study: SP ≥ 95%		≤ 2.6	68.5	96.6	98.0	54.9	19.86	0.33		76.5
[24]		≤ 8.0	97.3	75.9	91.0	91.7	4.04	0.04		91.2
This study: best SE and SP	≥ 30	≤ 2.8	84.3	91.7	95.2	75.0	10.11	0.17	0.937 ± 0.029	86.8
This study: SP ≥ 95%		≤ 1.75	70.0	97.2	98.0	62.5	25.20	0.31		79.2
[24]		≤ 4.2	95.7	69.4	85.9	89.3	3.13	0.06		86.8

The cut-offs that identify with a SP ≥ 95% those patients who should not undergo rhGH substitution therapy, because more probably not affected by GHD were: (i) 2.6 µg/l in overweight subjects with a SE and SP value of 68.5% and 96.6%, respectively; (ii) 1.75 µg/l in obese subjects with a SE and SP value of 70.0% and 97.2%, respectively (Fig. 2, Table 2). For lean subjects the cut-off identified at the first evaluation (i.e., 8 µg/l) was already characterized by a SP ≥ 95%.

The PPV, NPV, the ROC AUC, LHR + , LHR-, and the diagnostic accuracy of these cut-off limits to GHRH + ARG are reported in Table 2.

The current GHRH + ARG cut-offs (i.e., ≤ 11.5 µg/l in lean, ≤ 8.0 µg/l in overweight, and ≤ 4.2 µg/l in obese subjects) applied to our study population clearly overestimated the GHD diagnosis (Table 2).

### Side effects

Administration of GHRH + ARG did not cause any relevant side effect except for face flushing observed in 48 patients.

## Discussion

To our knowledge this is the first study that identifies the diagnostic cut-offs of GH response to GHRH + ARG related to BMI using a clinical definition of GHD as gold standard.

Our results, based on a considerable group of patients with a history of hypothalamic–pituitary disease, suggest that, with this new approach, the GHRH + ARG cut-offs should be revised. Indeed, our data specifically show that:

- The best cut-off to GHRH + ARG test for the diagnosis of adult GHD is 8.0 μg/l (SE 95%, SP 100%) in lean, 7.0 μg/l in overweight (SE 97.3%, SP 82.8%) and 2.8 μg/l (SE 84.3%, SP 91.7%) in obese subjects.
- However, in order to minimize the bias in GHD diagnosis, by reducing the false positive value (i.e. SP ≥ 95%), the cut-off should be further reduced to 2.6 μg/l (SE 68.5%, SP 96.6%) in overweight, and to 1.75 μg/l (SE 70.0%, SP 97.2%) in obese subjects.
- The current cut-offs to GHRH + ARG test for the diagnosis of adult GHD overestimate the diagnosis of GHD in either lean, overweight, and obese subjects.

The diagnosis of adult GHD is challenging for the clinician because of the lack of a single biological endpoint. Adult GHD diagnosis depends on the demonstration of a reduced peak serum GH level in response to one or more GH stimulation tests [2, 10, 11]. Testing for adult GHD should only be considered if there is a clinical suspicion of GHD and the intention is to treat if the diagnosis is confirmed [2, 10, 11]. Currently, there is no ideal stimulation test and the decision to consider performing a GH stimulation test to diagnose adult GHD must take into account the validity of the chosen test, its GH cut-offs, the availability of local resources, and the clinician expertise.

All GH stimulation tests are based on the concept that a pharmacological agent stimulates pituitary GH secretion, with peak GH levels detectable by timed frequent serum sampling after administration of the stimulus.

Initially, the diagnostic cut-offs for different stimulus tests were identified by looking for the minimal response observed in a group of normal subjects [33–35]; subsequently new studies have revisited the diagnostic criteria comparing the response to the test in analysis with that of the test considered the gold standard [21, 25–29], often comparing patients with normal subjects [22–24, 27, 28].

All these approaches are exposed to possible bias. In particular, the studies that compare the response of a group of patients already diagnosed for the target condition with the response observed in healthy volunteers, even if matched by sex, age and BMI, are at risk of overestimating the diagnostic accuracy of the cut-offs thus identified and, consequently, the results obtained cannot be applied to the clinical setting. On the other hand, the studies that establish the diagnostic accuracy of a particular test, comparing it with another diagnostic test, are at risk of overestimating or underestimating the diagnosis similarly to what the gold standard itself does.

Taking this into account, our work is the first that tried to identify the best cut-offs to GHRH + ARG test for the diagnosis of adult GHD using only a large group of patients, which is the most numerous reported to date in the literature; furthermore, in order to avoid the bias previously mentioned, we used a clinical criteria as gold standard; in particular we considered the presence of at least 3 other pituitary deficits as equivalent to the diagnosis of GHD and the absence of other hormonal deficits, together with an IGF-I SDS ≥ 0, as equivalent to the exclusion of GHD. Indeed, as reported in literature and in current guidelines [2, 36–38], the presence/absence of other pituitary deficits and the IGF-I SDS values are the two most important parameters for predicting a final diagnosis of GHD. These features provide valuable information that can be used as a proxy for estimating the probability of GHD prior to stimulation tests.

The correct choice of the clinical gold standard seems to be confirmed by the clinical differences between GHD and noGHD observed at baseline: it is not surprising that hypopituitary patients were those who more frequently presented a morpho-structural alteration of the hypothalamic-pituitary region or who more frequently had undergone neurosurgical and/or radiotherapy treatment [39–42]. Moreover, the higher prevalence of overweight and obese subjects in GHD group is in line with the finding that hypopituitary patients are usually characterized by an alteration in body composition similar to that of the metabolic syndrome [43]. Finally, our work confirms that the GH response to GHRH + ARG is significantly more compromised in patients with three or more pituitary hormone deficiencies than in patients without deficiencies [37].

Our work highlights one more time the well-known negative association between the GH response to stimulation tests and the BMI [21, 22, 24, 26, 27, 44] with a clear decline in the identified cut-off in lean, overweight and obese subjects. The distinct and progressive reduction of GH response to any stimulation test has clinical impact when these tests are used to demonstrate the presence of GHD [45]. Indeed, one of the warnings in interpreting the results of all the GH stimulation tests in adults is the high prevalence of obesity in the general population, as well as in patients with hypothalamic-pituitary disease [19]. Even if, to date, GHRH + ARG has already well established cut-off BMI related for the diagnosis of adult GHD, it should be considered that the previous study has evaluated the GH response to GHRH + ARG by comparing the response of patients who are certainly GHD with normal subjects [24].

As shown in Table 2, the new GH cut-off limits to GHRH + ARG, defined as the one with the best SE and SP, are characterized by a diagnostic accuracy that is better than the one of the previous cut-offs; in particular, the identified cut-offs generally improve SP maintaining a near comparable SE in lean and overweight subjects, while SE declines in obese subjects. Moreover, we decided to maximize SP to value ≥ 95% in the three BMI category groups, in order to correctly identify true GHD subjects, avoiding overtreatment in patients not actually GHD, particularly in obese population. With this new approach the cut-offs thus identified obviously reduce the SE values but are characterized by a marked increase in LHR + values ranging between 19.86 in overweight and 95 in lean subjects. The reduced diagnostic accuracy observed with this approach is clearly due to the reduction in the ability to identify some true GHD patients, but, on the other hand we can observe an improvement in the ability to rightly identify subjects that do not need GH replacement therapy.

We are also aware that the definition of a preserved somatotropic function used in this study (no other pituitary deficits and IGF-I SDS ≥ 0) results in a possible underestimation of isolated GHD cases. However, a previous study [46] has demonstrated that the absence of other pituitary deficiencies, along with IGF-I SDS levels > − 0.52 indicates a low pretest probability of GHD; in the current study, we aimed to further emphasize this concept by rising the threshold of IGF-I SDS to values ≥ 0. This adjustment was made to increase confidence in the clinical definition of no-GHD, ensuring that subjects identified as such truly have a low pretest probability of GHD.

However, considering the cost of rhGH replacement therapy, the potential side effects, and the lack of reliable data on strong end points, we believe that for GHD it is preferable to be sure to exclude from the therapy patients not affected by GHD, rather than not to treat patients with GHD. Considering this, the results of this study allow us to suggest the use of the new cut-offs with the best compromise between SE and SP (i.e. 8.0 μg/l, 7.0 μg/l, and 2.8 μg/l, in lean, overweight and obese subjects, respectively) in patients with a high pretest probability to be GHD, and the use of new cut-offs with SP ≥ 95% (i.e. 8.0 μg/l, 2.6 μg/l, and 1.75 μg/l, in lean, overweight and obese subjects, respectively) in patients with a low pretest probability.

Finally, it must be underlined that the cut-offs identified are acceptable for the assay used in this study; however, it must be emphasized that our study makes it possible to carry out a Bland–Altman plot to identify any correction factors for any other assay.

In conclusion, the results of this study, based on a considerable group of patients with a history of hypothalamic–pituitary disease and on a clinical gold standard, suggest that the GH cut-offs to GHRH + ARG test should be revised in all BMI thresholds, in order to avoid falsely positive diagnoses of severe GHD in adults.

It will need to be evaluated in future studies whether the lack of effectiveness of rhGH therapy on strong endpoints, such as increased mortality, could be partially attributed to the incorrect prescription of therapy to patients who have been identified as having GHD based on current cut-offs, but who do not meet the criteria for GHD according to the new cut-offs proposed by us.

## Data Availability

The data sets generated during and/or analyzed during the current study are not publicly available but are available from the corresponding author on reasonable request.
